# The effect of a serious game on empathy and prejudice of psychology students towards persons with disabilities

**DOI:** 10.4102/ajod.v8i0.328

**Published:** 2019-03-28

**Authors:** Linda Olivier, Paula Sterkenburg, Esmé van Rensburg

**Affiliations:** 1 School of Psychosocial Behavioural Sciences: Psychology, North-West University, Potchefstroom, South Africa; 2 Department of Clinical Child and Family Studies, Vrije Universiteit Amsterdam, Amsterdam, the Netherlands; 3 Amsterdam Public Health research institute (APH), Amsterdam, the Netherlands; 4 Bartiméus, Doorn, the Netherlands

## Abstract

**Background:**

Much has been done regarding the promotion of equality in rights in terms of legislation, but persons with disabilities remain marginalised in society. Negative attitudes and prejudice contribute towards numerous challenges for persons with disabilities.

**Objectives:**

This study investigated the level of empathy and prejudice of students towards persons with disabilities, and the effect of the use of a serious game to enhance empathy and reduce prejudice.

**Method:**

A randomised controlled experiment with pre-test, post-test and follow-up test was used. Availability sample (*N* = 83) of psychology university students (22% males; 78% females) was divided into an experimental group that played the serious game *The World of Empa* and two control groups. The first control group received texts on case studies and information on reacting in a sensitive and responsive way, and the second control group received no intervention.

**Results:**

Participants have average levels of empathy (score: 32 to 52) and strong prejudice (score: 0.08 to −0.87) towards persons with disabilities. The intervention results in a slight short-term effect for prejudice and sub-scale measurements of empathy. A slight improvement was noted in participants’ ability to transpose themselves imaginatively into the experience of disabled characters.

**Conclusion:**

The findings contribute to the understanding of empathy as a dynamic component that can be positively influenced by, for example, a serious game. These results have teaching implications on the facilitation of empathy. The short-term effect on empathy and prejudice towards persons with disabilities may contribute to bridge the inequality experienced by persons with disabilities.

**Keywords:**

empathy; prejudice; serious games; persons with disabilities; psychology students.

## Introduction

During the 1990s, South Africa experienced transformation on various levels, and even brought forth a new constitution that aimed to liberate the rights of persons with disabilities – enabling them to compete more equitably for health care funding and raising awareness for their needs (Kromberg et al. 2008). South Africa reached new frontiers by being one of a few countries to include disability issues in their constitution by enshrining civil and political rights for persons with disabilities, as well as rights concerning everyday existence, such as education, health, housing and social assistance (Heap, Lorenzo & Thomas 2009). Heap et al. (2009:859) reported that much has been done in protecting the rights of persons with disabilities in terms of legislation and administrative measures, but the reality of the implementation of these rights is not yet experienced in the day-to-day experiences of South African people with disabilities. Although the rights of persons with disabilities are strongly advocated in legislation, they remain disregarded in mainstream society as in South Africa, in 2011, only 1.8% of the persons with a disability were employed (Maja et al. 2011). Wiggett-Barnard (2013) also reports how persons with disabilities are under-represented in South African companies despite the various legislations and policies aimed to promote employment equality. Thus, the equality of rights for persons with disabilities are still far removed from the reality as persons with disabilities perform far worse on several indicators of living conditions in both high- and low-income countries, where they are often marginalised (Loeb et al. 2008).

## The challenges associated with disability

According to the World Health Organisation (WHO 2016), disability is a complex phenomenon referring to impairments, activity limitations and participation restrictions reflected in the interaction between the features of a person’s body and the features of that person’s environment. Even though definitions aim to explain disability and the various types, it is important to recognise that disability represents a range of medical and social conditions and a heterogeneous population, and failing to understand this diversity leads to stereotypical views that can negatively influence attitudes towards persons with disabilities (Wiggett-Barnard 2013). Negative attitudes towards persons with disabilities also contribute to negative and demeaning behaviour patterns such as acting in an aggressive or hostile way, talking to someone with a disability in a patronising way or staring at them (Aiden & McCarthy 2014). As a marginalised group, the accessibility to basic services in South Africa specifically also seems to be a problem to persons with disabilities (Heap et al. 2009).

Numerous studies indicate that there are concerns regarding South African health care relating to ineffectiveness, barriers to care and unmet health needs because of an unequal and unsuccessful health system (Goudge et al. 2009; Ruff et al. 2011; Xaba, Peu & Phiri 2012). Persons with disabilities are therefore further constrained because of the fact that their needs are not met, thus making it difficult for them to effectively function as their non-disabled peers in society. Studies indicate that persons with disabilities experience health inequalities caused by obstacles such as discriminatory attitudes that they face in accessing effective health care services (Melville 2005:124). Negative attitudes towards individuals with disabilities can therefore cause serious social and psychological problems without the health care professional even realising it. Furthermore, negative attitudes towards persons with disabilities are so general that it might not always be noticed by people without disabilities – leaving persons with disabilities feeling marginalised by society because of barriers that inhibit their lives (Johnson 2011). These negative attitudes can lead to feelings of loneliness, fear, isolation, the need for acceptance and various other psychosocial problems.

The need for psychological help, because of mental health problems, is therefore just as great for persons with disabilities as for persons without disabilities. Research shows that learning disabilities (Silver 1981; Taggart, Cousins & Milner 2007), hearing impairment (Danermark 1998; Fellinger et al. 2008; Van Eldik 2005), intellectual disabilities (Alimovic 2013; Carvill & Marston 2002; Gagliardi et al. 2011; Taggart, Taylor & McCrum-Gardner 2010), spasticity (Nicolson & Anderson 2001) and visual impairment (Eckerle et al. 2014; Pinquart & Pfeiffer 2014) are all related to the development of secondary emotional and behavioural problems. Unfavourable life events amongst persons with disabilities also increase the risk of psychological problems (Vereenooghe & Langdon 2013:4086). In addition, children with developmental disabilities are more at risk for the development of emotional and behavioural problems compared to their peers without a disability (Mazzucchelli & Sanders 2011:2148). Thus, psychologists can expect to provide psychological services to persons with disabilities with problems similar to those reported by persons without disabilities who seek help (Leigh et al. 2004). Empathy can be considered as an important construct in providing psychological services.

### Empathy

Persons with disabilities value principles such as acceptance and empathy within the psychotherapeutic approach (Johnson 2011). Empathy refers to the capability of being affected by and to share in another’s emotional state, and assessing possible reasons for their state to identify with the person by adopting his or her point of view (Burks & Kobus 2012). Leijssen (2004) also highlights the understanding of other as an essential element in helping relationships, and defines empathy as getting to know another, trying to understand them and their situation and reacting fittingly. According to Eriksen and McAuliffe (2006:180), empathy is seen as one of the key characteristics of student counsellors to become effective counsellors. For a therapist working with a client with a disability, this means that the therapist should move beyond the negative attitudes and stereotypes found in society towards trying to truly empathise and understand the context of the person with a disability

Unfortunately, it seems as if empathy can deteriorate as clinicians gain experience in the health service (Neumann et al. 2009). This negative relationship between empathy and experience was also seen in a study amongst psychology students at St. John’s University in the USA (Camarano 2011). In a profession such as psychology, empathy is considered a crucial value in psychotherapy with persons with disabilities (Johnson 2011). Considering the negative correlation between experience and empathy, as well as the importance of empathy in therapy, this study will focus on empathy and the enhancement thereof in third year and senior psychology students as these students might work in health services or continue their studies to become possible counsellors or psychologists.

### Prejudice

One of the barriers often cited as contributing to health inequalities experienced by persons with disabilities concerns the personal attributes (e.g. discriminatory attitudes, and a lack of appropriate knowledge and skills) of health care professionals (Melville 2005). Although staff attitudes towards persons with disabilities who exhibit behavioural challenges may crucially and negatively impact a positive support culture, very little research has focused on how held prejudice and negative attitudes can be changed (Hutchinson et al. 2014). Persons with impairments are restricted because of stigmatisation, prejudice and isolation from services. It is therefore apparent that interventions should be developed with goals to change staff attitudes towards persons with disabilities. This can be achieved with the use of appropriate teaching techniques relevant to the learning environment.

### Teaching and the role of serious gaming

In addressing the educating of psychology students, it is important to consider the learning context. Not only is it vital to integrate the needed knowledge and skills for working with persons with disabilities, but it is also important to address it in the appropriate learning environment. Currently, students function in an environment dominated by technology (e.g. computers, tablets, smart phones and social media). Serious gaming is one such modern teaching technique that has recently gained the attention of a wide variety of fields, where games can be used for professional teaching and not just for pure amusement (Breuer & Bente 2010). Serious games can be defined as games (or game-like interactive systems) with an engaging, self-reinforcing context to motivate and educate the players (Kankaanranta & Neittaanmäki 2008). It can be of any genre, use any game technology and be developed for any platform. Incorporating serious gaming into student teaching will stimulate learning.

There are various applications of related, and sometimes overlapping, fields such as e-learning, edutainment games and game-based learning that fall within the field of digital gaming (Susi, Johannesso & Backlund 2007). Serious games, however, move away from the negative stigma associated with the term ‘educational games’, as they not only focus on the educational aspects, but also place equal emphasis on pedagogy, simulations and the game (Ulicsak 2010). Distinct from educational games, serious games have a broader potential than merely addressing aspects from a curriculum, and they can reach adult audiences as well (Breuer & Bente 2010). Serious games therefore are used for purposes other than pure enjoyment by incorporating pedagogy and simulations into the game to reach a broader audience (Ulicsak 2010).

According to Blumberg, Blades and Oates (2013), research frequently highlights the negative impact of computer games without recognising the many advantages. Other than skills development and problem-solving abilities, one of the biggest advantages in the use of a serious game is the opportunity it gives students to experience situations that might be difficult or impossible to get in reality because of practical implications (Susi et al. 2007). The benefits of serious gaming can therefore be used for the education of psychology students in order to address prejudice and empathy towards persons with disabilities by giving them the exposure to the para-social interaction, the illusion of face-to-face relationship, with persons with different disabilities, which have been found to have a positively effect on the attitude to minority groups (Harwood 1999; Schiappa, Gregg & Hewes 2005).

*The World of Empa* (Sterkenburg 2012) is a serious game focused on the care of persons with disabilities. The player encounters a number of characters, namely a blind boy, a girl with multiple disabilities, a father, mother, baby and a boy with no disabilities. It is a computer game comprising six levels of principal educational situations, and questions are asked about the foremost educational and interaction problems encountered by the characters. Multiple answers are given as possibilities and if players respond by choosing an option reflecting an empathic and sensitive attitude, they are rewarded with points and move on to the next level. If the players do not respond empathically and sensitively, they lose points and must try again to complete the level. Throughout the game, players can see their score and if the game is finished, players receive feedback on their measure of sensitivity, empathy and responsiveness. The goal of the game is to discover the effect of sensitive and empathic reactions to situations (Sterkenburg 2012).

## Aims of the study

This study aimed to investigate the level of South African third year and senior psychology students’ empathy and prejudice, as well as how it might be influenced with the use of the serious game by investigating the following four research questions: (1) What is the level of empathy amongst third year and senior psychology students towards persons with disabilities? (2) What is the level of prejudice amongst third year and senior psychology students towards persons with disabilities? (3) What is the effect of *The World of Empa* on the third year and senior psychology students’ empathy towards persons with disabilities? (4) What is the effect of *The World of Empa* on the third year and senior psychology students’ prejudice towards persons with disabilities? We expect that psychology students will have a higher level of empathy and lower level of prejudice than the general population. As these third year and senior psychology students study to possibly become therapists, and empathy is an important characteristic of a therapist, we expect that these participants will have higher empathy and less prejudice than the general population. Furthermore, our hypothesis is that there will be a higher level of empathy after playing the serious game and that the students will have less prejudice after playing the game compared to the control groups.

## Method

### Research design

This quantitative study consisted of a randomised controlled experiment with a pre-test, immediate post-test and follow-up design. The study consists of one experimental group and two control groups. The experimental group played the serious game *The World of Empa*. The first control group read case studies with case studies and background information entitled ‘Attachment’ (Sterkenburg, Janssen & Schuengel 2010) with the same theoretical foundation as the serious game. The participants could not continue with the post-test without actually playing the game or reading the texts. The second control group received no intervention. All three groups were exposed to the pre-test and immediate post-test, as well as a follow-up post-test about a month later. Both control groups received access to the game after the follow-up measure.

### Participants and context

The study was conducted at the North-West University’s Faculty of Health Sciences, and specifically the subject groups psychology of the Potchefstroom and Vaal Triangle campuses, South Africa, in collaboration with the Vrije Universiteit Amsterdam, the Netherlands. Once approval from the involved universities was gained, departmental heads and lecturers involved with the third year and senior classes of the psychology subject groups at the Potchefstroom campus and the Vaal Triangle campus were identified as gatekeepers through whom prospective participants were contacted during a period of 2 months. Senior psychology students were defined as being third year and honours students. Non-probability sampling was used and participants were contacted through recruitment of volunteers in the relevant psychology classes on the Potchefstroom campus and the Vaal Triangle campus of the university. It was important that participants were fluent in English because of the nature of the serious game and measuring instruments. Initially, 100 senior students indicated interest in participation, but 17 potential participants withdrew because of transport problems, difficult academic schedules and semester tests. A total of 83 participants therefore participated in the study. Using electronic systematic random sampling, the participants were randomly divided between the experimental and control groups via electronic systematic randomisation. In 22 cases, the follow-up measure was missing. These missing values were scattered over the three conditions.

### Data collection and measuring instruments

After providing informed consent, participants were required to complete a demographic questionnaire that included questions regarding age, gender, race and level of education. Questions were asked to determine if the participant had a disability themselves or if they knew someone with a disability and the relation between them. These variables were examined and added as a confounder where necessary. Data were collected using the following validated questionnaires as measuring instruments with both the experimental and control groups:

The empathy quotient (EQ) is a self-reporting, quantitative instrument consisting of 60 items where participants must indicate on a four-point scale whether they agree or disagree with a specific statement, such as: ‘I am quick to spot when someone in a group is feeling awkward or uncomfortable’ (Billington, Baron-Cohen & Wheelwright 2007). A total EQ score of 32–52 indicates an average score, while 53–63 is above average and 64–80 indicates an understanding of how others feel and how to respond in a sensitive and empathic manner. The EQ measures different aspects of empathy on a cognitive and affective level and it has a high test–retest reliability measured over a period of 12 months (*r* = 0.97, *p* ≤ 0.001) and a high internal consistency (α = 0.92). The EQ was also shown to have concurrent validity (Lawrence et al. 2004).

The interpersonal reactivity index (IRI) is a quantitative measurement consisting of 28 items, such as ‘Before criticizing somebody, I try to imagine how I would feel if I were in their place’ (Davis 1980). There are four sub-scales (perspective taking, fantasy, empathic concern and personal distress) to assess multiple cognitive and affective components of empathy. Cognitive dimensions consist of perspective taking and fantasy, while affective dimensions include empathic concern and personal distress (Hawk et al. 2013). This frequently used self-report instrument is based on a multidimensional conceptualisation of empathy and is designed to assess individual differences in empathic tendencies (De Corte et al. 2007). The IRI has demonstrated good intra-scale and test–retest reliability, and convergent validity is indicated by correlations with other established empathy scales (Davis 1980).

The implicit association test (IAT) of Greenwald, McGhee and Schwartz (1998) seeks to measure implicit attitudes by measuring the underlying automatic evaluation, as it assesses the association between a target-concept discrimination (e.g. ‘handicapped’ and ‘no handicap’) and an attribute dimension (e.g. pleasant and unpleasant). It measures the implicit social preferences of participants using a categorical computer task where the strongest association between concepts is measured through reaction time (Karpinski & Steinman 2006). The IAT shows reliability in measuring the implicit cognitions amongst adults with a reliability value of α > 0.75 (Greenwald, Nosek & Banaji 2003). According to Bluemke and Friese (2008), flexibility, reliability and validity are acknowledged as valuable features of the IAT.

After completing the pre-test questionnaires, the Mersenne Twister pseudo-random number generator (PRNG) (Matsumoto & Nishimura 1998) automatically assigned the participant to one of the three conditions. As the process was automatically programmed, the researcher was blind to the condition of the participants.

### Data analysis

All data were analysed by the Statistical Consultation Services of the university, Potchefstroom campus, using a software program ‘Statistical Package for the Social Sciences’ standard version 22.0.1, 2014 (Field 2009). To achieve research aims 1 and 2, descriptive statistics, frequencies, means and standard deviations were used to analyse the data. According to Steyn et al. (1994), the mean is the best measure of locality to give an indication of the central tendency, while the standard deviation gives information concerning the distribution of the individual values around the mean.

The *d*-scores were calculated for the IAT according to the steps described by Greenwald et al. (2003). A *t*-test was performed to define the effects of demographical variables. Analysis of variance (ANOVA) repeated measures were used with the three assessments (T0 and T1 and T2) as ‘Time’ and ‘Condition’ (EMPA vs. case studies) as between factor. The intervention effect was examined as the main effect of condition and the Time × Condition interaction effect. The use of this analysis also enables the handling of missing data where not all participants took part in all three measurements.

### Ethical considerations

The Faculty of Health Sciences Research Ethics Committee evaluated and approved the merits of this study and ethical approval (NWU-00125-11-A1) was granted. Informed consent forms were completed by the participants, stipulating the details of the study and their voluntary participation in the project would not receive any form of compensation other than a certificate of participation, which was emailed to them.

## Results

The participants (*N* = 83) were randomly and electronically divided into the following three groups: an experimental group (*n* = 26), a control group that received a text to read (*n* = 26) and a control group that received no intervention (*n* = 31). Table 1 provides an overview of the demographical information of participants, indicating that the majority of participants (78%) were female with the majority of participants ranging from the ages of 18–21 years (49%) and 22–25 years (39%). Race was indicated by 60% of participants as Caucasian, 31% Africans and a mere 9% being of mixed race. No significant differences between the two groups were found for gender and age.

**TABLE 1:** Overview of frequency distribution of participants’ demographic information (*N* = 83).

Variable	Description	Frequency	Percentage
Gender	Male	18	22
	Female	65	78
Age	18–21 years	41	49
	22–25 years	32	39
	26+ years	10	12
Ethnicity	Caucasian	50	60
	African	26	31
	Mixed race	7	9
	Other	0	0
Experience working	None	70	84
with persons with	1–2 years	8	10
disabilities	3–5 years	2	2
	5+ years	3	4
Participants having	No	80	96
a disability (himself or herself)	Yes	3	4
Participants that	None	51	62
have family	Immediate	6	7
member(s) with disability	Extended	26	31
Participants that	None	46	55
know people other	Neighbour	12	15
than family that	Fellow student	21	25
have a disability	Other	4	5
Amount of contact	N/A	1	3
with person with	1–2 times a year	6	16
disability (*n* = 37)	1–2 times a month	10	27
	1–2 times a week	10	27
	Daily basis	10	27

*N*, population size; *n*, sample size; N/A, not applicable.

For the purpose of this study, participants’ contact and experience with persons with disabilities or their own disabilities were also examined. Only three of the participants had a disability themselves: two individuals reported having attention deficit hyperactivity disorder (ADHD) and one as having a hearing impairment. The vast majority of participants (84%) had no experience working with persons with disabilities. Exposure to persons with disabilities seemed limited with only 38% of participants having a family member with a disability or knowing any other person with a disability (45%). The extent of contact the 37 participants had with a non-related person with disabilities was noted as follows: noted as not applicable (*n* = 1), once or twice a year (*n* = 6), once or twice a month (*n* = 10), one to two times a week (*n* = 10) and a daily basis (*n* = 10).

The sub-scales of the IRI (fantasy, perspective taking, empathic concern and personal distress) were addressed separately during data analysis for the purpose of reliability. According to Field (2009), the Cronbach’s alpha should be applied separately to the sub-scales if the used questionnaire has sub-scales. The Cronbach’s alpha estimates the reliability by determining the internal consistency of the test or the average correlation of items within the test (Nunnally & Bernstein 1994). Table 2 gives an indication of the reliability of the measurements with overview of the Cronbach’s alpha of the EQ and sub-scales of empathy measured in the IRI. Although the general accepted Cronbach’s alpha value of 0.8 is considered appropriate for cognitive tests, values below even 0.7 can realistically be expected in the measurement of psychological constructs because of the diversity of the constructs being measured (Field 2009).

**TABLE 2:** Reliability of scales and sub-scales as well as descriptive statistics measured before any intervention.

Scales and sub-scales	Cronbach’s alpha	Mean	Standard deviation
EQ	0.82	46.57	10.40
IAT	N/A	−0.84	0.35
Fantasy	0.58	3.02	0.54
Perspective taking	0.59	3.79	0.63
Empathic concern	0.60	3.85	0.70
Personal distress	0.53	3.23	0.76

EQ, empathy quotient; IAT, implicit association test; N/A, not applicable.

As indicated in Table 2, a strong sense of reliability and internal consistency (Cronbach’s alpha = 0.82) was seen in the EQ. All the sub-scales of the IRI, however, showed a poor internal consistency with four items on the various sub-scales that had to be excluded in order to promote internal consistency. The items that negatively influenced the internal consistency of scales were removed before continuing further. The IAT only gives one reaction time score and has no questions and sub-scales. Determining the reliability was therefore not possible.

Table 2 also gives an indication of the level of empathy and prejudice as indicated by the means scored on the pre-test. Slightly low to average empathy levels were noted on the EQ (mean = 46.57), while the IRI sub-scales (measured on five-point scales) indicated moderate levels of empathy (means > 3.00). Participants’ level of prejudice showed strong negative associations with (strong negative associations towards) persons with disabilities (mean = −0.84) before any intervention.

The demographics did not show significant associations with the data, except for gender. Gender was the only demographical factor that showed significance influence on empathy scores as indicated in Table 3. On the EQ, women obtained a higher mean value of 49.25 comparing to the mean value of 39.88 of the men, resulting in a *p*-value of 0.01, and Cohen’s *d* = 1.03. Empathic concern was also higher in women (mean = 4.03) as compared to men (mean 3.39) (*p* = 0.01; *d* = 0.78). Fantasy and prejudice were unaffected by gender differences. No statistical differences were found relating to ethnicity, age or experience of disability.

**TABLE 3:** Results from *t*-test for gender comparisons on scores obtained before intervention.

Scales and sub-scales	Gender	*n*	Mean	Standard deviation	*p*	Effect size (Cohen’s *d*-score)
EQ	Male	16	39.88	8.71	0.01	1.03
	Female	67	49.25	9.10		
IAT	Male	16	−0.95	0.31	0.18	0.34
	Female	63	−0.82	0.37		
Fantasy	Male	16	2.89	0.52	0.18	0.37
	Female	67	3.09	0.55		
Perspective taking	Male	16	3.53	0.66	0.06	0.54
	Female	67	3.88	0.60		
Empathic concern	Male	16	3.39	0.82	0.01	0.78
	Female	67	4.03	0.58		
Personal distress	Male	16	2.95	0.47	0.19	0.50
	Female	67	3.31	0.72		

EQ, empathy quotient; IAT, implicit association test; N/A, not applicable; *n*, sample size.

Tables 4 and 5 provide an overview of the statistical and practical differences between groups, as well as over time. Although Tables 4 and 5 should be considered together, they are presented separately for clarity and ease of reading. When comparing the experimental group with the two control groups over time, all three groups were relatively comparable with average scores on empathy measured by the EQ (Table 4). Three statistical differences interaction effects between the experimental and control groups were found. Regarding the perspective taking sub-scale of the IRI control group 1 (CL) showed a decrease from the first time (mean = 4.14) to the third measurement (mean = 3.84). This resulted in main effects of Time × Condition for Perspective Taking (*F*(102.8) = 3.07, *p* = 0.02). Regarding empathic concern, the reading group (CL), showed an average decline of 4.11–4.01 over the three measurements. Repeated measures ANOVAs indicated a main effects of the Time × Condition interaction for Empathic concern (*F*(105.8) = 3.63, *p* = 0.01). There was no main effect of the Time × Condition for the IRI sub-scale fantasy (*F*(115.6) = 1.67, *p* = 0.16). Although not statistically significant, the experimental group as well as the CL group did show a medium practical significant decrease (*d* = 0.49 and 0.43, respectively) in the fantasy sub-scale of the IRI on the immediate post-test, while the control group receiving no intervention showed no significant changes over time (Table 5). The control group required to read did, however, score practically higher than the other groups on the first measurement of all IRI sub-scales but showed a medium practical significant decline on the follow-up measurement a month later (Table 5). Repeated measures ANOVAs indicated that a main effect of the Time × Condition interaction for personal distress was significant (*F*(105.7) = 3.46, *p* = 0.01), as the average score in the reading control group (CL) increased but then decreased again.

**TABLE 4:** Results of repeated measures analysis of variance with means between groups over time.

												Hierarchical linear models results								
Scales and sub-scales	E means			CL means			C means			MSE	Variance	Group			Time			Group & Time		
	Time^1^	Time^2^	Time^3^	Time^1^	Time^2^	Time^3^	Time^1^	Time^2^	Time^3^		Subject	df	*F*	*p*	df	*F*	*p*	df	*F*	*p*
EQ	46.00	44.18	44.97	49.02	50.12	49.05	47.23	45.66	48.26	17.2	91.4	81.5	1.13	0.33	98.7	125	0.78	98.7	0.770	0.55
Fantasy	2.96	3.17	2.91	3.31	3.22	3.09	2.93	3.08	3.10	0.17	0.11	102.3	1.18	0.31	115.7	0.65	0.53	115.6	1.670	0.16
Perspective taking	3.62	3.57	3.66	4.14	4.19	3.84	3.74	3.80	3.87	0.12	0.28	87.6	3.36	0.04	102.8	0.39	0.68	102.8	3.070	0.02*
Empathic concern	3.58	3.57	3.73	4.38	4.11	4.01	3.79	3.71	3.89	0.14	0.23	91.8	5.69	0.01	105.8	0.78	0.46	105.7	3.630	0.01*
Personal distress	2.98	2.99	3.12	3.72	3.85	3.35	3.08	3.25	3.27	0.19	0.22	92.6	6.93	0.00*	105.8	0.46	0.63	105.7	3.460	0.01*
IAT	−0.86	−0.81	−0.76	−0.87	−0.46	−0.75	−0.84	−0.65	−0.75	0.08	0.05	97.3	0.79	0.46	100.8	5.09	0.01*	100.8	0.962	0.43

EQ, empathy quotient; IAT, implicit association test; MSE, mean square error; E, experimental group; CL, control group (reading); C, control group (no intervention); numerator df (degrees of freedom) group = 2; numerator df time = 2; numerator group & time = 4; *, *p* < 0.05.

**TABLE 5:** Cohen’s *d* effect sizes indicating practical significant changes within groups over time for means in Table 4.

Variable	Experimental group			Control group (reading) (CL)		Control group (no intervention) (C)	
	Time	Time¹	Time²	Time¹	Time²	Time¹	Time²
EQ	Time²	0.18		0.11		0.15	
	Time³	0.10	0.08	0.00	0.10	0.10	0.25
Fantasy	Time²	0.40		0.19		0.29	
	Time³	0.09	0.49	0.43	0.25	0.32	0.03
Perspective taking	Time²	0.07		0.09		0.10	
	Time³	0.07	0.14	0.48	0.57	0.21	0.11
Empathic concern	Time²	0.01		0.44		0.13	
	Time³	0.25	0.26	0.61	0.17	0.16	0.29
Personal distress	Time²	0.01		0.19		0.25	
	Time³	0.21	0.20	0.59	0.77	0.30	0.04
IAT	Time²	0.15		1.12		0.51	
	Time³	0.27	0.12	0.33	0.78	0.24	0.27

EQ, empathy quotient; IAT, implicit association test; CL, control group (reading); C, control group (no intervention).

All three groups also showed comparable levels of prejudice with strong negative associations regarding persons with disabilities on the pre-test (means in Table 4). The negative value indicated a strong association between constructs of disability and unpleasantness. The closer the value is to 0, the less prejudice towards persons with disabilities. Repeated measures ANOVAs indicated no main effects of the Time × Condition interaction for IAT (prejudice) (*F*(100.8) = 3.96, *p* = 0.43). However, a significant effect of time (*F*(100,8) = 5.0, *p* = 0.01) was found, indicating an increase from pre-test to post-test and a decrease to follow-up, because of the declines in the post-test found in both control groups.

## Discussion

Empathy and acceptance play an important role in the therapeutic work with persons with disabilities who seek intervention for secondary psychological problems caused by the limitations experienced in their daily lives (Johnson 2011). Unfortunately, a negative correlation exists between therapists’ empathy and their gaining of experience (Camarano 2011). Attention is drawn to the possibility of influencing these components by using modern teaching techniques to gain experience and to increase the empathy to support the therapeutic work with persons with disabilities. The main objective of this study therefore was to examine the level of empathy and prejudice of third year and senior psychology students (as possible future therapists or health care workers) towards persons with disabilities and to study the use of a serious game to enhance empathy and reduce prejudice towards persons with disabilities by focusing on four underlying aims.

The first research aim was to explore the level of empathy amongst third year and honours psychology students towards persons with disabilities. The results from the EQ and sub-scales of IRI depicted students as having average levels of empathy towards persons with disabilities. The findings indicated no significant differences from demographical factors such as ethnicity, race and age on their level of empathy towards persons with disabilities. It is, however, noted that female participants scored higher on EQ (*p* = 0.01; *d* = 1.03), and empathetic concern (*p* = 0.01) with large effect (*d* = 0.78). Regarding perspective taking, there was a medium effect (*d* = 0.54); the *p*-value shows a trend (*p* = 0.06). This conclusion resonates with findings in research indicating gender differences in empathy with females tending to be more empathic (Baron-Cohen & Wheelwright 2004; Davis 1980). Gender was also noted to influence participants’ empathy sub-scales in the research conducted by Collins et al. (2015). It is, however, concerning, and not consistent with our expectation, that psychology students (as possible future counsellors or psychologists) only show an average level of empathy as they are in their preparation of possibly working in a profession that places high regard on empathic interpersonal relationships. Further research on the level of empathy found amongst psychology students might therefore be of value in the facilitation of empathy in students’ education curriculum to become psychologists.

The second research question focused on level of prejudice amongst third year and honours psychology students towards persons with disabilities. According to Banse, Seise and Zerbes (2001), the IAT is a reliable, valid measurement of implicit attitudes, but reliability can be influenced by procedural variations. To prevent procedural variations influencing reliability, all groups were exposed to the same procedure in identical conditions throughout pre-test, post-test and the follow-up. Results from the first measurement of the IAT indicate a comparable level of strong prejudice amongst all three groups with means averaging around *d-*scores of about −0.85. The results echo the moderate to strong negative attitudes towards persons with a disability found across 13 international studies that employed the IAT (Wilson & Scior 2014). The prejudice towards persons with disabilities can be because of the limited exposure the group, as full-time students, has with persons with disabilities. Social psychology strongly supports Allport’s contact hypothesis, claiming that one crucial means of reducing intergroup prejudice is through contact between groups (Pettigrew & Tropp 2006). This could possibly explain why the students with such low levels of exposure and experience in contact with persons with disabilities (Table 1) have high levels of prejudice towards persons with disabilities. This resonates with previous studies finding contact to account for significant differences in IAT scores (Wilson & Scior 2014). This result has significant implications for the consideration of practical exposure to minority groups such as persons with disabilities to address levels of prejudice in the curriculum of psychology students.

Thirdly, the study investigated whether *The World of Empa* could affect the psychology students’ empathy towards persons with disabilities. Significant main effects were found for perspective taking, empathetic concern and personal distress. However, changes seemed to occur in the control groups rather than in the experimental group (serious game). In fact, this may indicate that the serious game did prevent a decline in empathy. As this short intervention prevents decline in empathy, this result may support the importance of interactive learning compared to passive reading or no intervention at all. Further research on the use of serious games to improve empathy is necessary. For example, research on the effect of a serious game that is longer than the 20 min, or which has more sessions of serious gaming over time.

A small increase in the sub-scale of fantasy in the immediate post-test of the experimental group was noted. The fantasy scale measures the tendency to be caught up in fictional stories and to imagine oneself in the same situations as fictional characters, with the tendency to transpose oneself imaginatively into feelings and actions of the fictional characters (Davis 1980). The increase in fantasy after exposure to the serious game proves the important dynamics of games, play and imagination to enhance learning in such online contexts (Thomas & Brown 2011). This also supports the effect of serious games challenging the imagination by creating a fantasy on extrinsic and intrinsic level to facilitate learning (Gunter, Kenny & Vick 2006). It would appear that the imaginative contact that people with disabilities had through exposure to the game might have had a small positive effect on the enhancement of the students’ empathy towards disabilities, even if only on the short term. Thus, possibilities to sustain this positive effect over time need to be investigated.

It seems as if the reading of literature findings and case studies of persons with disabilities had a short-term effect on the emotional component of empathy. The four sub-scales of the IRI measure both cognitive dimensions (fantasy and perspective taking) and emotional dimensions (empathic concern and personal distress) of empathy as multidimensional components (Davis 1980). The reading of the text had a slight short-term decrease in the emotional dimension. This might indicate the factual text not appealing to the emotional dimension of the reader. None of these changes was, however, sustained over time. This temporary impact seems to support the notion that empathy as an embedded construct can be influenced in some degree but that it cannot be completely forced (Davis 1990). Hatcher et al. (1994) present research on the teachability of empathy using the IRI and they, however, suggest that a developmental sequence exists for the four sub-scales of the IRI and that future studies might need to sample larger populations in early adulthood, non-college and culturally diverse populations in order to gain clarity on this matter. Further research on the possibility of empathy as a teachable construct is therefore needed on a larger scale over a longer period to gain clarity of the effect of intervention strategies with the aim of enhancing empathy.

Finally, our hypothesis was that the level of prejudice towards persons with disabilities would decline when third year and senior psychology students’ played the serious game *The World of Empa*. No significant changes were noted within the experimental group regarding prejudice towards persons with disabilities. Although Crisp and Turner (2009) provide empirical research indicating that even imaginary intergroup interactions can support the contact hypothesis to reduce prejudice, it seems as if the aimed imaginary exposure in simulated contact through the serious game did not have a significant effect. This might be because of the lack of frequency as the contact hypothesis implies that increased contact with out-groups can lead to a decline in prejudiced attitudes (Beelmann & Heinemann 2014). In the context of the current study, however, participants were just exposed to the simulated contact with persons with disabilities through the serious game on a single occasion, which might not have been enough to be significant. The possibility of increased frequency in simulated contact in order to reduce prejudice with a serious game might be considered in future studies.

## Theoretical and practical implications

The results of the current study not only hold implications for future research considerations, but also hold theoretical and practical repercussions. The average levels of empathy noted, and limited research on the level of empathy amongst psychology students, indicate the need for future research in order to promote the theoretical understanding of empathy and its facilitation in practice. Small short-term effects seen in the results might support the notion that empathy is a dynamic component. Theoretically, more research might be needed to determine the dynamics behind empathy in order to settle the debate in the literature about empathy as a teachable construct or not. The slight short-term effects noted on reducing prejudice confirm the theory of contact hypothesis that increased contact with persons with disabilities might be needed to challenge negative attitudes and that once-off simulations cannot be enough to change the deep-rooted stereotypical societal beliefs towards persons with disabilities.

These findings also have practical implications for the teaching curriculum of psychology students as attention might be needed to address their exposure and frequency of contact with persons with disabilities. Careful consideration should also be given throughout the curriculum to facilitate empathy as a dynamic component. This can be accomplished with possible use of reflection in supervision, increased interaction with persons with disabilities in practical exposure as well as experiential learning.

## Limitations and future recommendations

During the process of this study, certain challenges contributed to limitations of the study. Because of the voluntary nature of the research, 22 participants did not return for the follow-up post-test, therefore creating incomplete data sets. However, the dropout was scattered over the experimental and control groups. To limit any technical problems, a technical assistant was present and supported the students at the start-up of the study. Nevertheless, three students mentioned that they had difficulty concentrating and felt frustrated because of the electronic data collection in a big computer room and some technical computer difficulties. The initial intention was to have a bigger sample, but because of limited interest and participants withdrawing, it was not possible. Furthermore, findings need to be interpreted in light of the possibility of type I errors. This places severe limitations on statistical justifications and the generalisation of the study. However, these findings are of great interest for psychology curriculum development for senior students at universities across South Africa. For a follow-up study, it is recommended that a bigger scale study should be considered.

The Cronbach’s alpha scores of the sub-scales were 0.58, 0.59, 0.6 and 0.53. These scores are low coefficients, however, adequate given the low number of items for each sub-scale. The low number of items was because of the fact that almost half of the items had to be excluded. These items were excluded from the IRI in order to promote internal consistency. They were reverse score items, which might indicate possible problems in participants’ interpretation of reverse score items. Although the IRI is regarded as one of the best measures of empathy developed, the IRI may measure processes broader than empathy as seen in some items included in sub-scales of fantasy and personal distress (Baron-Cohen & Wheelwright 2004). This might also have an impact on the reliability of the IRI’s depiction of students’ empathy levels.

The possibility of the questionnaires (poor internal consistency of the IRI) and game developed in the European context having an impact on the results within the South African context cannot be excluded. It would be interesting to see if there are any differences between the study of the *Vrije Universiteit*, Amsterdam (which developed the game and started with the same study in the Netherlands) and results found within the South African context to investigate the possibility of cultural influences on empathy and prejudice. Therefore, a comparative study between the current study and the results from the same study conducted in the Netherlands might be recommended. With the negative correlation between experience and empathy as described in the literature, it can also be considered to investigate the possible interventions in enhancing empathy within an older population. The current study focused on third year and honours psychology students with limited work experience and exposure to the demands of practical care. Focusing future studies on an older population with more experience can be considered to determine if intervention might be more effective once empathy has already started declining with the gaining of experience.

## Conclusion

The aim of this study was to investigate psychology students’ level of empathy and prejudice towards persons with disabilities. Furthermore, the use of a serious game to influence empathy and prejudice towards persons with disabilities was examined. The results indicate that the ability to enhance empathy and reduce prejudice towards persons with disabilities might have a positive contribution to bridge the equality promoted in legislations to the experienced day-to-day reality of persons with disabilities. Short-term effects with regard to the use of the serious game and informative text, in contrast with no changes in the control group, support the possibility of empathy being facilitated. However, future research efforts are needed in the consideration of establishing possible long-term changes in society, and particularly regarding the psychotherapeutic work with persons with disabilities within the South African context.

## References

[CIT0001] Aiden H. & McCarthy A., 2014, *Current attitudes towards disabled people*, Scope, pp. 1–20, viewed 28 October 2018, from http://www.scope.org.uk

[CIT0002] Alimovic S., 2013, ‘Emotional and behavioural problems in children with visual impairment, intellectual and multiple disabilities’, *Journal of Intellectual Disability Research* 57(2), 153–160. 10.1111/j.1365-2788.2012.01562.x 22563696

[CIT0003] Banse R., Seise J. & Zerbes N., 2001, ‘Implicit attitudes towards homosexuality: Reliability, validity, and controllability of the IAT’, *Zeitschrift für Experimentelle Psychologie* 48, 145–160. 10.1026//0949-3946.48.2.145 11392982

[CIT0004] Baron-Cohen S. & Wheelwright S., 2004, ‘The empathy quotient: An investigation of adults with Asperger syndrome or high functioning autism, and normal sex differences’, *Journal of Autism and Developmental Disorders* 34(2), 163–175. 10.1023/B:JADD.0000022607.19833.00 15162935

[CIT0005] Beelmann A. & Heinemann K.S., 2014, ‘Preventing prejudice and improving intergroup attitudes: A meta-analysis of child and adolescent training programs’, *Journal of Applied Developmental Psychology* 35(1), 10–24. 10.1016/j.appdev.2013.11.002

[CIT0006] Billington J., Baron-Cohen S. & Wheelwright S., 2007, ‘Cognitive style predicts entry into physical sciences and humanities: Questionnaire and performance tests of empathy and systemizing’, *Learning and Individual Differences* 17(3), 260–268. 10.1016/j.lindif.2007.02.004

[CIT0007] Bluemke M. & Friese M., 2008, ‘Reliability and validity of the Single-Target IAT (ST-IAT): Assessing automatic affect towards multiple attitude objects’, *European Journal of Social Psychology* 38(1), 977–999. 10.1002/ejsp.487

[CIT0008] Blumberg F.C., Blades M. & Oates C., 2013, ‘Youth and new media: The appeal and educational ramifications of digital game play for children and adolescents’, *Zeitschrift für Psychologie* 221(2), 67–71. 10.1027/2151-2604/a000133

[CIT0009] Breuer J. & Bente G., 2010, ‘Why so serious? On the relation of serious games and Learning’, *Eludamos Journal for Computer Game Culture* 4(1), 7–24.

[CIT0010] Burks D.J. & Kobus A.M., 2012, ‘The legacy of altruism in health care: The promotion of empathy, prosociality and humanism’, *Medical Education* 46(1), 317–325. 10.1111/j.1365-2923.2011.04159.x 22324531

[CIT0011] Camarano V., 2011, ‘Examining emotional and cognitive empathy in psychology graduate Students’, *Dissertation Abstracts International* 71, 7748.

[CIT0012] Carvill S. & Marston G., 2002, ‘People with intellectual disability, sensory impairments and behaviour disorder: A case series’, *Journal of Intellectual Disability Research* 43(3), 264–272. 10.1046/j.1365-2788.2002.00400.x 11896812

[CIT0013] Collins K., Gratton C., Heneage C. & Dagnan D., 2015, ‘Employed carers’ empathy towards people with intellectual disabilities: The development of a new measure and some initial theory’, *Journal of Applied Research in Intellectual Disabilities* 30, 133–146. 10.1111/jar.12226 26514549

[CIT0014] Crisp R.J. & Turner R.N., 2009, ‘Can imagined interactions produce positive perceptions? Reducing prejudice through simulated social contact’, *American Psychologist* 64(4), 231–240. 10.1037/a0014718 19449982

[CIT0015] Danermark B.D., 1998, ‘Hearing Impairment, emotions and audiological rehabilitations: A sociological perspective’, *Scandinavian Audiology* 27(1), 125–131. 10.1080/010503998420757 10209787

[CIT0016] Davis C.M., 1990, ‘What is empathy, and can empathy be taught?’, *Physical Therapy* 70(11), 707–711. 10.1093/ptj/70.11.707 2236214

[CIT0017] Davis M.H., 1980, ‘A multidimensional approach to individual differences in empathy’, *JSAS Catalogue of Selected Documents in Psychology* 10, 85.

[CIT0018] De Corte K., Buysse A., Verhofstadt L.L., Roeyers H, Ponnet K. & Davis M.H., 2007, ‘Measuring empathic tendencies: Reliability and validity of the Dutch version of the Interpersonal Reactivity Index’, *Psychologica Belgica* 47(4), 235–260. 10.5334/pb-47-4-235

[CIT0019] Eckerle J.K., Hill L.K., Iverson S., Hellerstedt W., Gunnar M. & Johnson D.E., 2014, ‘Vision and hearing deficits and associations with parent-reported behavioural and developmental problems in international adoptees’, *Maternal Child Health Journal* 18(1), 575–583. 10.1007/s10995-013-1274-1 23605963 PMC4161370

[CIT0020] Eriksen K.P. & McAuliffe G.J., 2006, ‘Constructive development and counsellor competence’, *Counsellor Education & Supervision* 45(3), 180–192. 10.1002/j.1556-6978.2006.tb00141.x

[CIT0021] Fellinger J., Holzinger D., Sattel H. & Laucht M., 2008, ‘Mental health and quality of life in deaf pupils’, *European Child & Adolescent Psychiatry* 17(7), 414–423. 10.1007/s00787-008-0683-y 18810312

[CIT0022] Field A., 2009, *Discovering statistics using SPSS*, 3rd edn., Sage, London.

[CIT0023] Gagliardi C., Martelli S., Tavano A. & Borgatti R., 2011, ‘Behavioural features of Italian infants and young adults with William-Beuren syndrome’, *Journal of Intellectual Disability Research* 55(2), 121–131. 10.1111/j.1365-2788.2010.01376.x 21205040

[CIT0024] Goudge J., Gilson L., Russel S., Gumede T. & Mills A., 2009, ‘Affordability, availability and acceptability barriers to health care for the chronically ill: Longitudinal case studies from South Africa’, *BMC Health Services Research* 9, 75 10.1186/1472-6963-9-75 19426533 PMC2694171

[CIT0025] Greenwald A.G., McGhee D.E. & Schwartz J.L.K., 1998, ‘Measuring individual differences in implicit cognition: The Implicit Association Test’, *Journal of Personality and Social Psychology* 74(6), 1464–1480. 10.1037/0022-3514.74.6.1464 9654756

[CIT0026] Greenwald A.G., Nosek B.A. & Banaji M.R., 2003, ‘Understanding and using the implicit association test: An improved scoring algorithm’, *Journal of Personality and Social Psychology* 85(2), 197–216. 10.1037/0022-3514.85.2.197 12916565

[CIT0027] Gunter G.A., Kenny R.F. & Vick E.H., 2006, ‘A case for a formal design paradigm for serious games’, *The Journal of the International Digital Media and Arts Association* 3(1), 93–105.

[CIT0028] Harwood J., 1999, ‘Age identification, social identity gratifications, and television viewing’, *Journal of Broadcasting and Electronic Media* 43, 123–136.

[CIT0029] Hatcher S.L., Nadeau M.S., Walsh L.K. & Reynolds M., 1994, ‘The teaching of empathy for high school and college students: Testing Rogerian methods with the Interpersonal Reactivity Index’, *Adolescence* 29(116), 961–974. 7892806

[CIT0030] Hawk S.T., Keijsers L., Branje S.J.T., Van der Graaff J., De Wied M. & Meeus W., 2013, ‘Examining the interpersonal reactivity index (IRI) among early and late adolescents and their mothers’, *Journal of Personality Assessment* 95(1), 95–106. 10.1080/00223891.2012.696080 22731809

[CIT0031] Heap M., Lorenzo T. & Thomas J., 2009, ‘We’ve moved away from disability as a health issue, it’s a human rights issue’: Reflecting on 10 years of the right to equality in South Africa’, *Disability & Society* 24(7), 857–868. 10.1080/09687590903283464

[CIT0032] Hutchinson L.M., Hastings R.P., Hunt P.H., Bowler C.L., Banks M.E. & Totsika V., 2014, ‘Who’s challenging who? Changing attitudes towards those whose behaviour Challenges’, *Journal of Intellectual Disability Research* 58(2), 99–109. 10.1111/j.1365-2788.2012.01630.x 23046106

[CIT0033] Johnson C., 2011, ‘Disabling barriers in the person-centred counselling relationship’, *Person-Centred and Experiential Psychotherapies* 10(4), 260–273. 10.1080/14779757.2011.626627

[CIT0034] Kankaanranta M. & Neittaanmäki P. (eds.), 2008, *Design and use of serious games*, vol. 37, Springer Science & Business Media, Dordrecht

[CIT0035] Karpinski N. & Steinman R.B., 2006, ‘The single category implicit association test as a measure of implicit social cognition’, *Journal of Personality and Social Psychology* 91(1), 16–32. 10.1037/0022-3514.91.1.16 16834477

[CIT0036] Kromberg J., Zwane E., Manga P., Venter A., Rosen E. & Christianson A., 2008, ‘Intellectual disability in the context of a South African population’, *Journal of Policy and Practice in Intellectual Disabilities* 5(2), 89–95. 10.1111/j.1741-1130.2008.00153.x

[CIT0037] Lawrence E.J., Shaw P., Baker D., Baron-Cohen S. & David A.S., 2004, ‘Measuring empathy: Reliability and validity of the empathy quotient’, *Psychology Medicine* 34(1), 911–924. 10.1017/S0033291703001624 15500311

[CIT0038] Leigh I.W., Powers L., Vash C. & Nettles R., 2004, ‘Survey of psychological services to clients with disabilities: The need for awareness’, *Rehabilitation Psychology* 49(1), 48–54. 10.1037/0090-5550.49.1.48

[CIT0039] Leijssen M., 2004, ‘Empathie als instrument voor effectieve geneeskunde’ [Empathy as an Instrument for effective medicine], in Leijssen M. & Stinckens N. (eds.), *Wijsheid in gesprekstherapie*, pp. 313–332, Leuven Universitaire Press, Belgium.

[CIT0040] Loeb M., Eide A.H., Jelsma J., Toni M.K. & Maart S., 2008, ‘Poverty and disability in Eastern and Western Cape provinces, South Africa’, *Disability & Society* 23(4), 311–321. 10.1080/09687590802038803

[CIT0041] Maja P.A., Mann W.M., Sing D., Steyn A.J. & Naidoo P., 2011, ‘Enlplying people with disabilities in South Africa’, *South African Journal of Occupational Therapy* 41(1), 24–32.

[CIT0042] Matsumoto M. & Nishimura T., 1998, ‘Mersenne twister: A 623-dimensionally equidistributed uniform pseudo-random number generator’, *ACM Transactions on Modeling and Computer Simulation (TOMACS)*, 8, 3–30.

[CIT0043] Mazzucchelli T.G. & Sanders M.R., 2011, ‘Preventing behavioural and emotional problems in children who have a developmental disability: A public health approach’, *Research in Developmental Disabilities* 32(6), 2148–2156. 10.1016/j.ridd.2011.07.022 21831592

[CIT0044] Melville C., 2005, ‘Discrimination and health inequalities experienced by disabled people’, *Medical Education* 39(1), 122–126. 10.1111/j.1365-2929.2004.02061.x 15679678

[CIT0045] Neumann M., Bensing J., Mercer S., Ernstmann N., Ommen O. & Pfaff H., 2009, ‘Analysing the ‘nature’ and ‘specific effectiveness’ of clinical empathy: A theoretical overview and contribution towards a theory-based research agenda’, *Patient Education and Counselling* 74(3), 339–346. 10.1016/j.pec.2008.11.013 19124216

[CIT0046] Nicolson P. & Anderson P., 2001, ‘The psychosocial impact of spasticity related problems for people with multiple sclerosis: A focus group study’, *Journal of Health Psychology* 6(5), 551–567. 10.1177/135910530100600508 22049453

[CIT0047] Nunnally J. & Bernstein I.H., 1994, *Psychometric theory*, 3rd edn., McGraw-Hill Inc., New York.

[CIT0048] Pettigrew T.F. & Tropp L.R., 2006, ‘Does intergroup contact reduce prejudice? Recent meta-analytic findings’, *Journal of Personality and Social Psychology* 90(5), 751–783. 10.1037/0022-3514.90.5.751 16737372

[CIT0049] Pinquart M. & Pfeiffer J.P., 2014, ‘Change in psychological problems of adolescents with and without visual impairment’, *European Child and Adolescent Psychiatry* 23, 571–578. 10.1007/s00787-013-0482-y 24132832

[CIT0050] Ruff B., Mzimba M., Hendrie S. & Broomberg J., 2011, ‘Reflections on health-care reforms in South Africa’, *Journal of Public Health Policy* 32, 184–192. 10.1057/jphp.2011.31 21730990

[CIT0051] Schiappa E., Gregg P.B. & Hewes D.E., 2005, ‘The parasocial contact hypothesis’, *Communication Monographs* 72(1), 92–115. https://doi.org/10.1080/0363775052000342544

[CIT0052] Silver L.B., 1981, ‘The relationship between learning disabilities, hyperactivity, distractibility, and behavioural problems: A clinical analysis’, *Journal of the American Academy of Child Psychiatry* 20(2), 385–397. 10.1016/S0002-7138(09)60996-1 7264110

[CIT0053] Steyn A.G.W., Smit C.F., Du Toit S.H.C. & Strasheim C., 1994, *Moderne statistiek vir die praktyk*, Van Schaik, Pretoria.

[CIT0054] Sterkenburg P.S., Janssen C. & Schuengel C., 2010, ‘Onderlinge verbondenheid: Begeleiding en zorg voor mensen met een verstandelijke en/of andere beperkingen’ [Interconnectedness: Guidance and care for persons with a mental and/or other Disability], viewed 15 April 2015, from http://bartimeus.nl/kennisbank/gehegtheid_3

[CIT0055] Sterkenburg P.S., 2012, ‘Serious gaming als interventie voor het stimuleren van sensitiviteit, empathie en responsiviteit van begeleiders in de zorg voor mensen met een visuele en/of verstandelijke beperking’ [Serious gaming as an intervention for the stimulation of sensitivity, empathy and responsiveness of facilitators in the care for persons with a visual and/or mental disability], in Zevalkink J. & Groenendijk I. (eds.), *Psychoanalyse en de media: Gebruik van media binnen en buiten de behandelkamer*, pp. 37–47, Van Gorcum, Assen.

[CIT0056] Susi T., Johannesson M. & Backlund P., 2007, ‘Serious games: An overview’, viewed 06 April 2015, from http://www.diva-portal.org/smash/get/diva2:2416/FULLTEXT01.pdf

[CIT0057] Taggart L., Cousins W. & Milner S., 2007, ‘Young people with learning disabilities living in state care: Their emotional, behavioural and mental health status’, *Child Care in Practice* 13(4), 401–416. 10.1080/13575270701488816

[CIT0058] Taggart I., Taylor D. & McCrum-Gardner E., 2010, ‘Individual, life events, family and socio-economic factors associated with young people with intellectual disability and with and without behavioural/emotional problems’, *Journal of Intellectual Disabilities* 14(4), 267–288. 10.1177/1744629510390449 21285121

[CIT0059] Ulicsak M., 2010, ‘Games in education: Serious games’, in *Futurelab*, viewed 09 August 2014, from http://www.futurelab.org.uk

[CIT0060] Thomas D. & Brown J.S., 2011, *A new culture of learning: Cultivating the imagination for a world of constant change*, CreateSpace, Lexington, Kentucky.

[CIT0061] Van Eldik T., 2005, ‘Mental health problems of Dutch youth with hearing loss as shown on the youth self report’, *American Annals of the Deaf* 150(1), 11–16. 10.1353/aad.2005.0024 15969220

[CIT0062] Vereenooghe L. & Langdon P.E., 2013, ‘Psychological therapies for people with intellectual disabilities: A systematic review and meta-analysis’, *Research in Developmental Disabilities* 34(11), 4085–4102. 10.1016/j.ridd.2013.08.030 24051363

[CIT0063] Wiggett-Barnard C., 2013, ‘Disability employment attitudes and practices in South African companies: A survey and case studies’, PhD dissertation, University of Stellenbosch, Stellenbosch.

[CIT0064] Wilson M.C. & Scior K., 2014, ‘Attitudes towards individuals with disabilities as measured by the Implicit Association Test: A literature review’, *Research in Developmental Disabilities* 35(2), 294–321. 10.1016/j.ridd.2013.11.003 24316588

[CIT0065] World Health Organisation (WHO), 2016, *Health topics: Disabilities*, viewed 10 September 2016, from http://www.who.int/topics/disabilities/en/

[CIT0066] Xaba N.A., Peu M.D. & Phiri S.S., 2012, ‘Perceptions of registered nurses regarding factors influencing service delivery in expanding programmes in a primary healthcare setting’, *Health SA Gesondheid* 17(1), viewed 05 February 2014, from 10.4102/hsag.v17i1.535.4

